# MET Exon 14 Skipping and Novel Actionable Variants: Diagnostic and Therapeutic Implications in Latin American Non-Small-Cell Lung Cancer Patients

**DOI:** 10.3390/ijms252413715

**Published:** 2024-12-22

**Authors:** Solange Rivas, Romina V. Sepúlveda, Ignacio Tapia, Catalina Estay, Vicente Soto, Alejandro Blanco, Evelin González, Ricardo Armisen

**Affiliations:** 1 Centro de Genética y Genómica, Instituto de Ciencias e Innovación en Medicina, Facultad de Medicina Clínica Alemana Universidad del Desarrollo, Santiago 7550000, Chile; i.tapial@udd.cl (I.T.); catalina.estay@mayor.cl (C.E.); vicente.soto@mayor.cl (V.S.); ablanco@udd.cl (A.B.); evefeliu@gmail.com (E.G.); 2 Center for Bioinformatics and Integrative Biology, Facultad de Ciencias de la Vida, Universidad Andres Bello, Av. República 330, Santiago 8370146, Chile; romina.sepulveda@unab.cl

**Keywords:** non-small-cell lung cancer, novel actionable variants, c-Met inhibitors, *MET* exon 14 skipping, next-generation sequencing

## Abstract

Targeted therapy indications for actionable variants in non-small-cell lung cancer (NSCLC) have primarily been studied in Caucasian populations, with limited data on Latin American patients. This study utilized a 52-genes next-generation sequencing (NGS) panel to analyze 1560 tumor biopsies from NSCLC patients in Chile, Brazil, and Peru. The RNA sequencing reads and DNA coverage were correlated to improve the detection of the actionable *MET* exon 14 skipping variant (METex14). The pathogenicity of *MET* variants of uncertain significance (VUSs) was assessed using bioinformatic methods, based on their predicted driver potential. The effects of the predicted drivers VUS T992I and H1094Y on c-MET signaling activation, proliferation, and migration were evaluated in HEK293T, BEAS-2B, and H1993 cell lines. Subsequently, c-Met inhibitors were tested in 2D and 3D cell cultures, and drug affinity was determined using 3D structure simulations. The prevalence of *MET* variants in the South American cohort was 8%, and RNA-based diagnosis detected 27% more cases of *METex14* than DNA-based methods. Notably, 20% of *METex14* cases with RNA reads below the detection threshold were confirmed using DNA analysis. The novel actionable T992I and H1094Y variants induced proliferation and migration through c-Met/Akt signaling. Both variants showed sensitivity to crizotinib and savolitinib, but the H1094Y variant exhibited reduced sensitivity to capmatinib. These findings highlight the importance of RNA-based *METex14* diagnosis and reveal the drug sensitivity profiles of novel actionable *MET* variants from an understudied patient population.

## 1. Introduction

Lung cancer is the leading cause of cancer-related death worldwide, and non-small-cell lung cancer (NSCLC) is the primary form of the disease, accounting for approximately 85% of cases. Lung cancer is often diagnosed at an advanced stage, making it challenging to provide curative therapy [1,2,3]. The treatments available for cancer patients before the advent of personalized medicine had a wide range of patient responses and relatively low survival [4]. The implementation of tyrosine kinase inhibitors has decreased NSCLC mortality since 2013 [5]. Nowadays, the American Society of Clinical Oncology (ASCO) and the European Society for Medical Oncology (ESMO) recommend sequencing actionable NSCLC genes, namely, *EGFR*, *ALK*, *ROS1*, *ERBB2*, *MET*, *BRAF*, *KRAS*, *RET,* and *NTRK1/2/3.* The identification of actionable genes has contributed to the molecular characterization of NSCLC [6,7]; however, due to the limited implementation of personalized medicine, some populations, such as Latin American NSCLC patients, have been poorly characterized [6].

Among the NSCLC actionable genes is *MET*, a critical oncogene that belongs to the transmembrane receptor tyrosine kinase family (RTK). The c-Met receptor is activated by the specific ligand hepatocyte growth factor (HGF) [7,8]. It induces receptor dimerization, followed by the c-Met autophosphorylation of three tyrosine residues (Y1230, Y1234, and Y1235) and a consequent increase in the c-Met tyrosine kinase activity [9,10]. The canonical c-Met signaling pathways include RAS/MAPK, which promotes a proliferative and survival phenotype [11], and the PI3K/Akt/mTOR signaling, which promotes survival; likewise, it has a migratory phenotype [11,12].

The juxtamembrane domain encoded by exon 14 acts as a binding site for the c-Cbl E3 ubiquitin ligase for protein degradation [13]. However, the c-Met receptor is constitutively expressed when exon 14 is missing due to different mutational events [14]. The *MET* exon 14 skipping variant (*METex14*), which occurs in 2–3% of NSCLC patients, is the only *MET* actionable variant recognized in NSCLC [15] and confers clinical sensitivity to c-Met inhibitors such as crizotinib [16], capmatinib [17], tepotinib [18], and savolitinib [19]. These c-Met inhibitors are currently considered the first line of therapy or a subsequent therapy option in patients with advanced or metastatic NSCLC [20,21].

*METex14* variants affecting the splicing region are challenging to diagnose since DNA sequencing has shown a low sensitivity [22,23]. Thus, sequencing tumor RNA is the current gold-standard method of diagnosis since it directly detects the fusion of exon 13 to exon 15, independent of the DNA variants that promote exon 14 skipping [24]. The variants that induce *METex14* represent a group of alterations at the 3′ splice site in intron 13 or the 5′ end splice site in intron 14, which involve donor or receptor sites and include single-nucleotide variants (SNVs) as well as insertions and deletions (INDELs) of different lengths in the splicing regions at the beginning and the end of exon 14 [15,25].

Diverse next-generation sequencing (NGS)-based protocols propose a threshold number of RNA reads necessary to diagnose *METex14*. For example, according to the user guide, the threshold for identifying *METex14* using an Oncomine^®^ assay is at least 120 fusion reads [26]. However, a study suggested a threshold of 800 reads to mitigate potential difficulties in interpretation [27]. However, the clinically relevant question that remains unaddressed beyond the bioinformatic threshold is as follows: how many RNA reads (or other appropriate metrics) are necessary to achieve a successful therapeutic response to MET inhibitors? [27,28].

In addition to the canonical actionable *METex14* variant, variants of unknown clinical significance (VUSs) are being increasingly reported in the tumor profile of NSCLC patients, which cannot be treated via targeted therapy. However, with the advent of new bioinformatic algorithms, the reclassification of these VUSs has allowed us to assertively determine if those protein changes could be an actionable variant. For instance, the Cancer Genome Interpreter (CGI) platform’s prediction algorithm helps identify driver variants that could potentially be treated with targeted therapies using BoostDM and OncodriveMut [29,30]. The Combined Annotation-Dependent Depletion (CADD) value is a pathogenicity predictor that integrates several genomic features and functional predictions [31]. The SIFT algorithm predicts whether an amino acid substitution is likely to affect protein function [32,33]. Polyphen-2 is another variant effect predictor that divides the analysis into three categories: benign, possibly damaging, and probably damaging to the protein function [34]. Johnson et al. recently demonstrated that at least 24% (106/438) of the VUSs analyzed were oncogenic, and, most importantly, they identified 44% (204/438) as potentially actionable variants [35]. Certain *MET* mutagenic events, such as variants affecting the juxtamembrane or the tyrosine kinase domains, can transform the c-Met into an oncogenic protein. Thus, the study and identification of novel *MET* actionable variants that impact these domains is essential to broaden the prescription of already-approved targeted drugs [13,36].

In this study, the analysis of the next-generation sequencing data of actionable genes from NSCLC South American patients demonstrated that 8% were *MET* variants, versus 4% in the GENIE cohort. So, we focused on the most prevalent *MET* variant, *METex14* (DNA versus RNA diagnosis) and the in vitro validation of two novel actionable variants categorized as VUSs. Identifying actionable and potentially actionable variants in under-represented populations is essential since, currently, actionable variants are identified primarily in Caucasian and non-Hispanic patients. Thus, identifying novel actionable variants in South American patients would reduce disparities in diagnosing actionable variants, expanding the benefits of the available targeted therapies to a broader patient population.

## 2. Results

### 2.1. The Mutational Profiles of NSCLC Actionable Genes in South Americans Evidenced a High Prevalence of MET Exon 14 Skipping and Uncertain Significance MET Variants

**Figure 1:**
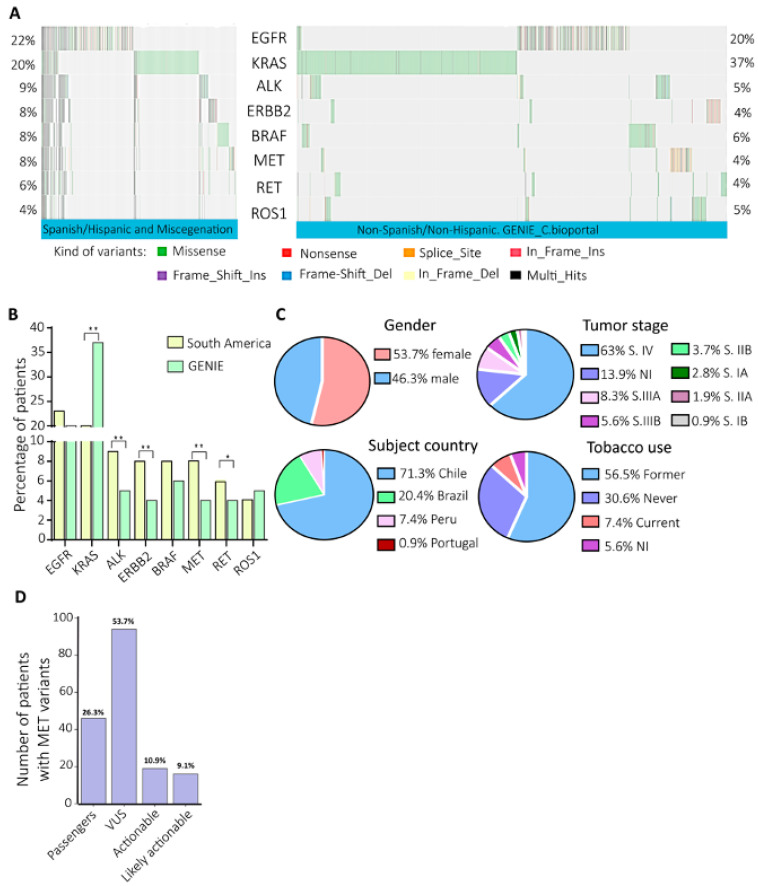
The mutational profiles of NSCLC actionable genes in South America evidenced a high prevalence of MET variants. (**A**) Each column of the oncoplots represents a patient, and the rows show the prevalence of the variants in the eight NSCLC actionable genes. (**B**) Comparison of variant prevalence in eight actionable NSCLC genes. (**C**) The gender, subject country, tumor stage, and tobacco use information of the patients with variants of the *MET* gene. (**D**) MET variant categorization according to the clinical significance, the number, and the percentage of the patients. * *p*-value ≤ 0.05; ** *p*-Value ≤ 0.01.

In the South American cohort considered in this study, protein-affecting variants (SNVs, indels, and gene fusions) in actionable NSCLC genes were discovered in 1560 patients (NCT03220230) [37,38]). The clinical information and the DNA prevalence of the variants were compared with a 100% non-Spanish/non-Hispanic Caucasian subset cohort of 18,857 NSCLC patients selected from GENIE [39] (Table S1 and Figure 1). *KRAS* was highly mutated in the Caucasians, compared to the South American cohort (37% vs. 20%). The genes *ALK* (9% vs. 5%), *ERBB2* (8% vs. 4%), and *MET* (8% vs. 4%) were significantly more mutated in the South American NSCLC cohort (Figure 1B). The high prevalence of *MET* variants in our cohort and the slow regulatory approval process of c-Met inhibitors in the region prompted us to focus the analysis on this particular gene.

#### Clinical Characteristics of the Patients with MET Variants

In the South American NSCLC cohort, 8% (123/1560) of the patients presented with *MET* variants: 53.7% were women and 46.3% were men (Figure 1C); 71.3% were from Chile, 20.4% from Brazil, and 7.4% from Peru (Figure 1C). The average age was 66 (Figure S1A). Almost all the patients with *MET* variants had one to three variants in any OFA gene (Figure S1B). At diagnosis, 63% of the patients were at stage IV of the disease (Figure 1C). Interestingly, 30.6% of the patients with *MET* variants had never smoked, and 52.5% said they were former smokers (Figure 1C).

The clinical significance categorization of the *MET* variants showed that 10.9% (19 patients) had known actionable *MET* variants, and 9.1% had likely actionable variants (12 patients). Diverse expert panels highly recommend the use of c-Metinhibitors for these patients, but the regulatory agencies do not recognize this form of treatment yet [40]. However, VUSs were the biggest category, accounting for 53.7% (92 patients) of the variants (Figure 1D).

### 2.2. The RNA and DNA MET Sequencing Analysis Evidenced Differences in Diagnosing the MET Exon 14 Skipping Variant

**Figure 2:**
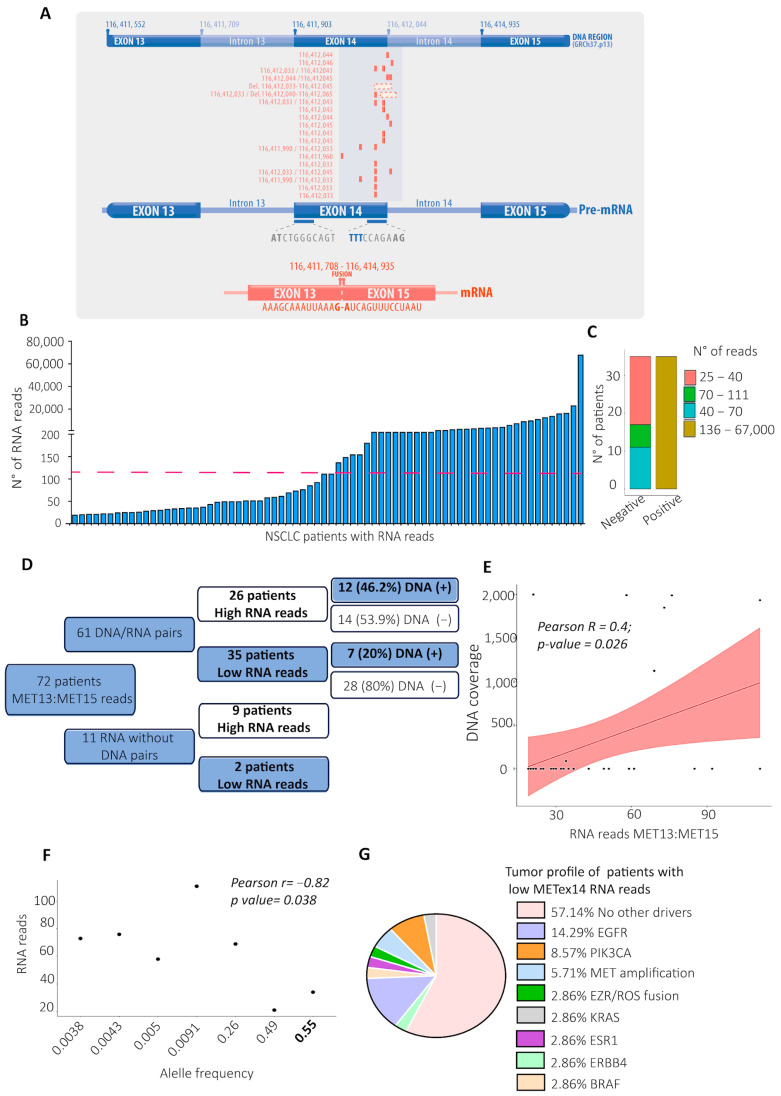
The RNA and DNA MET sequencing analysis evidenced differences in diagnosing the MET exon 14 skipping variant. (**A**) DNA regions of exons 13, 14, 15, and introns 14 and 15 of the MET gene (GRCh37.p13). Below are the DNA variants’ locations, which affect the coding sequence of exon 14 and the splicing donor region of the MET gene (variants located in red in rows) of each patient with a DNA variant in the SD region. (**B**) The broad spectrum of the RNA reads for the METex14 variant is shown in the x-axis. Each column represents a patient; the dashedline shows the threshold (120 reads) for the positive *METex14* diagnosis [26]. (**C**) The number of patients categorized as negative and positive for *METex14,* according to the numbers of RNA reads. (**D**) The conceptual map represents all TBx from the NSCLC patients with a pair of RNA- and DNA-sequenced QC pass data. (**E**) The Pearson correlation between the RNA and DNA reads is represented by a continous line and the standard error as a shadow. (**F**) The Pearson correlation analysis between the allele frequency of the positive *METex14* DNA variants (X-axis) and the number of RNA reads (Y-axis). (**G**) Altered genes in the tumor profile of the patients with low RNA reads for *METex14*.

Nineteen DNA samples that were positive for *METex14* variants had SNVs and deletions in the region of the splice donor (SD) site, and, interestingly, the analysis of these tumor biopsies (TBx) showed up two SNVs at the splice region of *MET* (Figure 2A, affected DNA regions in red). In the RNA analysis, 72 patients showed between 20 and 67.000 RNA reads for the fusion of exons 13 and 15 (Figure 2B). However, 48.6% (35/72) of the patients were confirmed positive for *METex14* (>120 RNA read threshold) and so were called the high-RNA-read subgroup. The 37 patients with fewer than 120 RNA reads were categorized as undetermined (low-RNA-read subgroup), following the advice of the Oncomine Focus Assay user guide [26], as depicted in Figure 2C.

Of the patients with an RNA diagnosis of *METex14*, 53.9% were negative for *METex14* in their DNA (Figure 2D), even in those with 3170—9487 and 12,301 RNA reads (Table S2). Here, the Pearson correlation between the DNA coverage and the RNA reads was R = 0.45 with a *p*-value of 0.02, where zero DNA reads mean the METex14 variant was not detected (Figure S2A). This moderate correlation indicates that in 45% of cases, an increase in RNA reads tends to increase the same unit in DNA reads.

Regarding the 35 patients with fewer than 120 RNA reads, 20% (seven patients) showed variants in their DNA, located in the SD region at the 5’ exon 14 (Figure 2A, the last seven DNA regions are in red, and Figure 2D). Here, the Pearson correlation between the low-RNA-read subgroup and their DNA coverage was R = 0.38, with a *p*-value = 0.026 (Figure 2E), which indicates a moderate correlation in 38% of cases (Figure S2A), showing that the DNA and RNA *METex14* diagnoses are moderately correlated, independently of the number of RNA reads.

Nevertheless, the allele frequencies (AFs) are unrelated to the number of RNA reads (Figure 2F). Some AFs could suggest germinal variants (AF around 0.5); unfortunately, the information on germline variants for these patients is unavailable. Notably, 57.14% of these patients with low RNA reads did not report other actionable variants in their tumor profiles for any targeted therapy indication for solid tumors. However, this percentage could increase to 71.43%, considering that *ESR1* (2.86%), *ERBB4* (2.86%), and *PIK3CA* (8.57%) are not actionable genes in lung cancer (Figure 2G).

### 2.3. MET T992I and H1094Y Were the Most Prevalent Predicted Driver Variants, Evidencing Biological Traits in Non-Tumor and NSCLC Cells Through the AKT Signaling Pathway

#### 2.3.1. VUSs Represented 53.7% of All the MET Variants (Figure 1C), So We Sought Those That Could Predict Actionability

**Figure 3:**
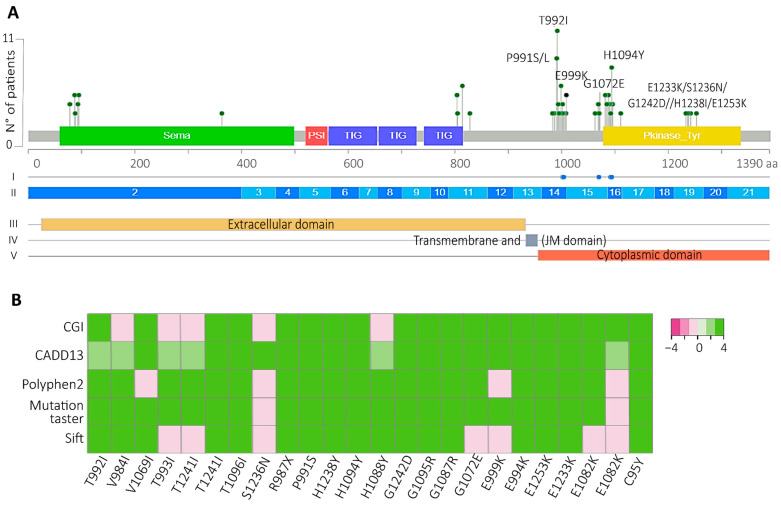
T992I and H1094Y were the most prevalent and bioinformatically predicted drivers and actionable. (**A**) All the VUSs were localized in the Met protein domains. The green, red, blue, and yellow rectangles represent the location of the Sema, PSI, TIG, and kinase protein domains, respectively. Above the lolliplot, (I) blue dots represent regions sensitive to targeted therapies, according to Oncokb. (II) The exons are represented by blue and light-blue boxes. (III-IV-V) The subcellular location of the mature protein. (**B**) The driver prediction of the VUSs located at the JM and TK domains (x-axis) using the bioinformatic algorithms CGI, Cadd13, polyphen2, mutation taster, and sift. Light pink and white represent predicted passengers and tolerated variants; green represents those variants’ predicted drivers.

Considering c-Met functional domains, we chose the variants that impacted the JM and the TK domains (Figure 3A). To gather more information about the driver statement of these VUSs, bioinformatic algorithms such as SIFT, mutation taster, and PolyPhen-2 were chosen to predict the potential effect of the variants on the protein function. Variants were categorized as tolerated (T), neutral (N), or deleterious (D). In addition, the CADD assigned a score between 0 and 40, where a high pathogenicity potential score was over 25. Finally, CGI predicted whether those VUSs were passengers and whether they were known or predicted actionable variants. Intriguingly, most of the variants in these domains were predicted to be potential drivers, as shown in the heatmap (Figure 3B and Table S3). The two most prevalent VUSs that were predicted to be drivers were T992I, which affected the JM domain (11 patients), and H1094Y, which affected the TK domain (6 patients). Additionally, 41.2% of the 17 patients with T992I and H1094Y did not have any other drivers or actionable variants in their tumor mutational profile (Figure S3). In total, 57.1% of these patients had no therapy option, since *PIK3CA* (14.3%), *IDH2* (7.1%), and *MTOR* (7.1%) are not actionable lung cancer genes.

#### 2.3.2. The VUSs Predicted to Be Drivers Induced a Migratory Phenotype and the Survival of Proliferative Cells in NSCLC and Non-Tumor Cells, Respectively

**Figure 4:**
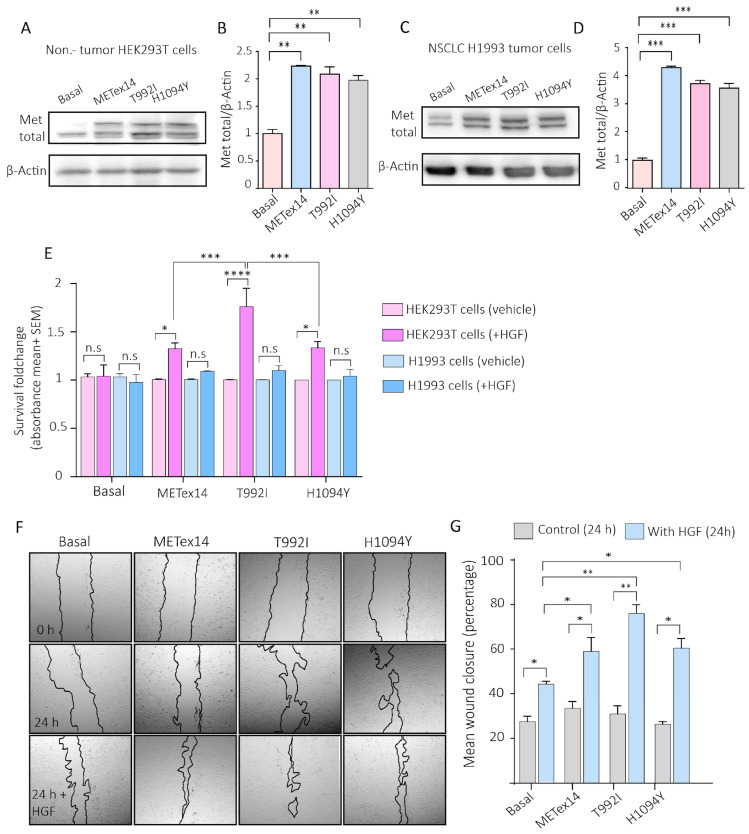
The VUSs predicted to be drivers, T992I and H1094Y, promote the survival of proliferative non-tumor cells and migration in tumor cells. (**A**) Representative Western blots of total Met and β-actin expression, evaluated for the H1993 GFP (basal), METex14, T992I, and H1094Y cells. (**B**) Densitometry levels of total normalized Met/β-actin (+SEM). The graph represents the normalized average from 3 independent experiments, ±SEM. (**C**) The representative Western blots of total Met and β-actin expression were evaluated for the HEK293T GFP (basal), METex14, T992I, and H1094Y cells. (**D**) Densitometry levels of total normalized Met/β-actin (+SEM). The graph represents the normalized average from 3 independent experiments, ±SEM. (**E**) The absorbance averages of HEK293T and H1993 cells expressing GFP, METex14, T992I, and H1094Y; the cells incubated with and without HGF. (**F**) Representative microphotographies of the wound healing at 0 and 24 h of H1993 cells expressing METex14, T992I, and H1094Y, treated with and without HGF were taken at 4×. (**G**) The wound closure percentage was calculated for each experimental condition. Finally, three independent experiments averaging the ±SEM are shown. A two-way ANOVA with Tukey correction was applied, and the *p*-values were adjusted for multiple comparisons. * *p* < 0.05; ** *p* < 0.01; *** *p* < 0.001; **** *p* < 0.0001; n.s. non-significant.

The expression of the T992I, H1094Y, and METex14 variants in non-tumor HEK293T cells, NSCLC H1993 cells, and BEAS-2B was confirmed using Western blots (Figure 4A–D and Figure S4). HEK293T cells expressing METex14, T992I, and H1094Y had increased survival in response to 24 h of HGF incubation, with T992I having significant survival regarding METex14 and the H1094Y variants. Additionally, the NSCLC H1993 cells did not change in response to HGF in any experimental condition (Figure 4E). However, these cells showed a high migration rate, estimated through the wound closure percentage in response to HGF (Figure 4F,G).

#### 2.3.3. The VUSs T992I and H1094Y Increased c-Met Autophosphorylation and Downstream Akt Activation

**Figure 5:**
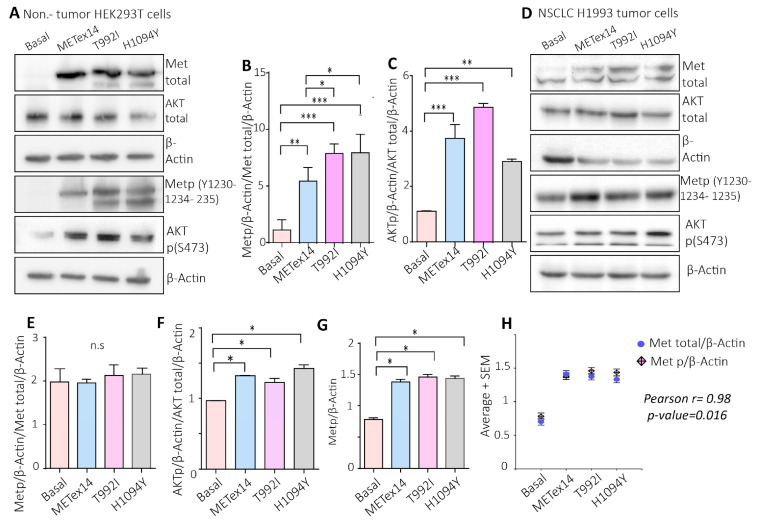
The VUSs predicted to be drivers, T992I and the H1094Y, increased Met-activating phosphorylation and the downstream Akt signaling pathway. (**A**) Representative Western blot images of total Met, total Akt, β-actin, Met p(Y1230-1234-1235), and Akt p(S473) protein expression of HEK293T cells. (**B**,**C**) The densitometry levels of Met phosphorylation, Akt phosphorylation, and β-actin were normalized relative to the total Met and Akt. (**D**) Representative Western blots of total Met, total Akt, β-actin, Met p(Y1230-1234-1235), and Akt p(S473) protein expression of H1993 cells. (**E**,**F**) Densitometry levels of Met phosphorylation, Akt phosphorylation, and β-actin normalized relative to the total Met and Akt for H1993 cells. Graphs represent the normalized average from 3 independent experiments, ±SEM. (**G**) Densitometry levels of Metp, relative to β-actin levels. (**H**) Pearson correlation between the Metp and Met total, relative to β-actin levels. A one-way ANOVA with Tukey correction was applied, and the *p*-values were adjusted for multiple comparisons. * *p* < 0.05; ** *p* < 0.01; *** *p* < 0.001; n.s. non-significant.

To know whether the variants induced migration and survival through c-Met activation, the protein extracts of the Hek293T cells expressing Metex14, T992I, and H1094Y were evaluated through Western blotting, evidencing a high level of c-Met-activating phosphorylation (Metp) and the activating phosphorylation of Akt (Aktp) (Figure 5A–C). However, the H1993 cells expressing METex14, T992I, and H1094Y did not show changes in Metp; nevertheless, the levels of the activating phosphorylation on Akt were significantly higher in the METexon14, T992I, and H1094Y conditions (Figure 5D–F). Still, we noticed high levels of Metp/β-actin in all the experimental conditions (Figure 5G), evidencing a proportional increase in Metp regarding the total expression of Met (Figure 5H). In addition, the bronchial–alveolar cells BEAS-2B also showed increased Metp and Aktp in METex14, T992I, and H1094Y compared with the basal expression (Figure S5A–D).

### 2.4. The VUSs Predicted to Be Driver Variants Were Sensitive to c-Met Inhibitors in 2D and 3D Cultures of Non-Tumor and Tumor Cells

**Figure 6:**
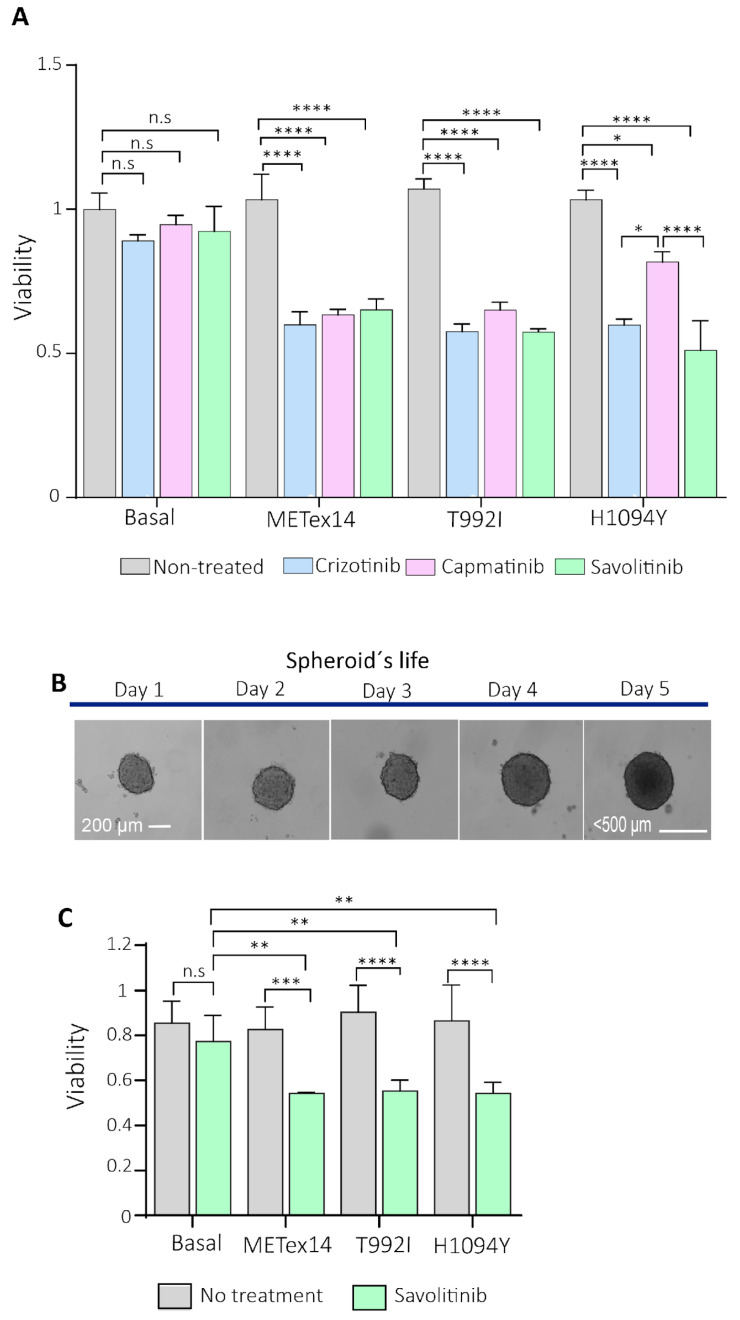
The 2D and 3D cell cultures expressing the Met-predicted driver variants were sensitive to c-Met inhibitors. (**A**) A total of 2000 HEK293T-expressing variants were seeded in 2D and incubated for 24 h with crizotinib, capmatinib, and savolitinib. The absorbance was calculated from three independent experiments and normalized, relative to the non-treatment culture cells. (**B**) Representative microphotographs (taken at 10×) were captured with a Cytation3 imaging reader of the 3D H1993 cells (spheroid) on each day of their life. On day 2 of spheroid formation (~200 µm sphere diameter), the drugs were incubated, and then the cells were released from the treatment until day 5. (**C**) The spheroids were treated with savolitinib for 24 h. Each experimental condition consisted of triplicates, averaged for each experimental condition. The three independent experiments were averaged, ±SEM. * *p* < 0.05; ** *p* < 0.01; *** *p* < 0.001; **** *p* < 0.0001 and n.s non-significant.

C-Met inhibitors, such as crizotinib, capmatinib, and savolitinib, were approved based on clinical trials that included patients whose tumors harbored METex14. Thus, we estimated the half-maximal inhibitory concentration (IC_50_) of crizotinib, capmatinib, and savolitinib in HEK293T cells expressing METex14 (Figure S6). Then, the cell response to the targeted treatments was evaluated, and we found that all treatments decreased the viability of cells expressing the METex14, T992I, and H1094Y variants compared to their counterpart untreated or control cells (Figure 6A). Furthermore, viability decreased in cells expressing the METex14 and T992I variants relative to their basal conditions when treated with crizotinib, capmatinib, and savolitinib (Table S4). On the other hand, cells expressing H1094Y showed a low sensitivity to capmatinib compared to the crizotinib and savolitinib treatments. Indeed, the capmatinib treatment did not significantly reduce cell viability relative to the basal conditions (Table S4).

#### 2.4.1. Savolitinib Treatment in Three-Dimensional (3D) Culture Cells (Spheres)

As previously recommended, the spheroids were allowed to grow for 120 h, not exceeding 500 microns in diameter [41] (Figure 6B). After 48 h of seeding, the spheres were treated with savolitinib for 24 h. After 48 h of drug release, the spheres expressing the METex14, T992I, and H1094Y variants showed the decreased viability of proliferating cells in response to savolitinib treatment, in comparison to the no-treatment condition, relative to the basal condition (Figure 6C), strongly suggesting that VUS T992I and H1094Y are actionable variants.

#### 2.4.2. Analysis of Atomic Interactions of Active Protein Met WT, METex14, T992I, and H1094Y Variants with Savolitinib Using Molecular Modeling, Docking, and Molecular Dynamics Simulations

**Figure 7:**
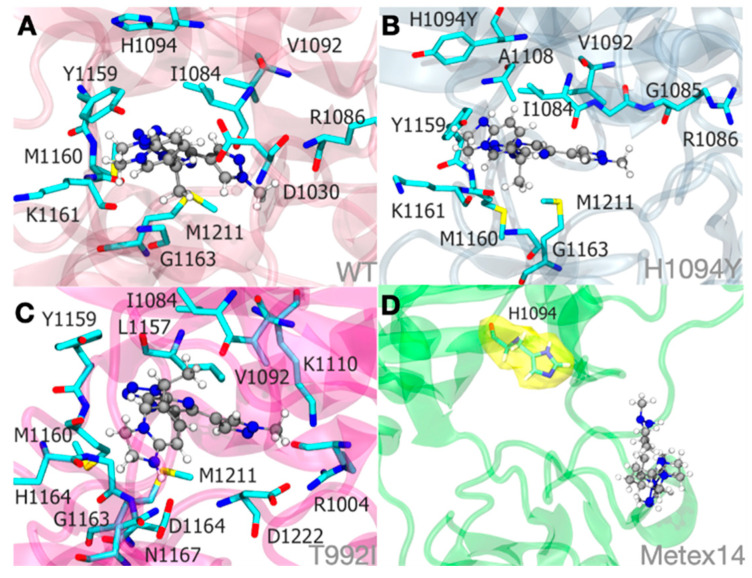
Molecular dynamics simulations illustrate the savolitinib–MET protein system binding at an approximate mean distance of 3.0. The stability of the pyridazinone ring of savolitinib within the binding site of the MET WT:SLB (**A**), MET H1094Y:SLB (**B**), and MET T992I:SLB (**C**) complexes is largely determined by hydrophobic interactions. Importantly, within the METex14:SLB complex (**D**), savolitinib is incapable of achieving a stable conformation due to substantial modifications in the initial loop that precedes the tyrosine kinase domain. Therefore, an unstable pocket site was produced. To clarify its proximity to savolitinib, the H1094 residue in the METex14:SLB complex (**D**) is highlighted in yellow in this context.

The models revealed a significant region lacking a secondary structure at the juxtamembrane domain, where the T992I variants are located. The secondary structure composition was analyzed along the trajectory, showing consistent levels of the alpha helix (23.8%) and beta sheet (14%) in the Met WT system. These proportions were maintained in the presence of savolitinib across different systems, including WT:savolitinib, T992I:savolitinib, and H1094Y:savolitinib. However, Metex14 displayed variability in its secondary structure composition (30.4% alpha helix/11.7% beta sheet), suggesting potential changes in the tertiary structure. The stability of savolitinib within the Met WT:savolitinib system was controlled by Y1159 (65% of the time) and M1169 (35% of the time) through a π-π interaction and hydrophobic contribution (Figure S7). Additionally, the interaction relied on the savolitinib pyrazolopyrimidine and D1030 (41% of the time) (Figure 7A), which explains the sensitivity. This was also observed in the Met H1094Y:savolitinib system, where Y1094 was directly in contact with savolitinib (Figure 7B). Concerning the T992I:savolitinib system, the average distance between the Cα T992I and the savolitinib was ~20 Å; therefore, T992I did not have direct contact with savolitinib. The T992I:savolitinib system induced a modification in the binding pocket, where the H1094 lost its π-π interaction positioning at around 8 Å from savolitinib (Figure 7C). Despite this, the savolitinib binding site remained due to D1164 (57% of the time) and M1169 (45% of the time). The METex14 system is composed of the juxtamembrane domain, which has not previously been defined by structural studies, and significantly disrupts the structure of the Met tyrosine kinase, which is unable to establish the same stable binding pocket for savolitinib (Figure 7D). For more information, you can download the videos on the drug–protein interactions (Supplementary Videos).

## 3. Discussion

Previously, 1881 DNA and RNA reads from South American NSCLC patients’ tumors were sequenced using the Oncomine Focus Assay (NCT03220230) [37]. An analysis of 1560 patients with DNA- and RNA-sequenced QC pass data showed a DNA MET variant prevalence of 8%, twice that which is reported in the Caucasian, non-Spanish/non-Hispanic patients from the GENIE cohort (Figure 1B) [39]. This finding is supported by data from the Caucasian and Asian NSCLC cohorts, which were found to have a range from 0.9% to 4.0% of MET variants using whole-exome sequencing and the Ion AmpliSeqTM Library Kit 2.0, respectively [42,43]. The patients with a high percentage of MET variants in the South American cohort were principally from Chile (71.3%), where MET inhibitors have not been approved yet [44]. On the other hand, Brazil has already approved the prescription of crizotinib, campatinib, and tepotinib [45], as has Argentina [46]. Nonetheless, comparisons of molecular epidemiology in different cohorts are not straightforward and must be interpreted cautiously.

The METex14 variant was the most prevalent diagnosis, and at the RNA level, 72 TBx from NSCLC patients presented an extensive range of RNA reads for this variant (25 to 67.000 RNA reads). However, of these 72 patients, 35 had more than 120 RNA reads, which is the number required for diagnosing METex14, according to the user guide of the Oncomine Focus Assay [26]. Interestingly, 53.9% of these RNA diagnoses were negative in the DNA sequencing, evidencing the high sensitivity of the METex14 RNA diagnosis, as reported before [23].

In addition, 37/72 patients had fewer than the 120 RNA reads required for METex14 detection; however, 20% of them had pathogenic DNA variants in the splicing region of MET with diverse allele frequencies (Figure 2A,G). The splicing region is often disrupted when a variant is present [47], confirming the possibility of false-negative METex14 diagnoses with fewer than 120 RNA reads. As an RNA sample from tumor biopsies is the gold standard for METex14 diagnosis, in cases with fewer than the required number of RNA reads indicated in the user guide of the NGS kit, it would be necessary to call these cases undetermined until the DNA sequencing or RT-PCR [48] brings new evidence.

While the number of METex14 RNA reads indicative of successful therapy with MET inhibitors continues to be unknown, it is essential to gather as much clinical and molecular evidence as possible for those cases with low RNA reads whose tumor profiles did not show actionable nor driver variants, such as the 57% of the patients with low RNA reads for METex14 analyzed here. Until now, the sole evidence for how many reads are required for successful therapy with c-Met inhibitors was a case report about the highest sensitivity of the OFA, which detected 46 reads for METex14 in an NSCLC patient without known actionable variants, who was previously treated with various chemotherapeutic agents without positive effects. The patient demonstrated an immediate therapy response after tepotinib, a c-Met inhibitor, was administered [28].

Another interesting finding was the high percentage of VUS-predicted drivers that impacted the JM and TK c-Met domains (Figure 3A), with H1094Y and T992I being the most prevalent, which affected 14.41% of the patients with Met variants in the South American cohort. The H1094Y variant was first identified in renal cell carcinoma [49,50], and it has been detected as a resistance variant after glesatinib treatment in NSCLC patients positive for METex14 [51]. Additionally, this variant has been associated with an oncogenic role in NSCLC in conferring acquired resistance to EGFR-TKIs [52,53]. Although savolitinib alone has not been approved by the FDA yet, the combination of savolitinib plus osimertinib for NSCLC patients with amplified MET or who are positive for EGFR has been successful (NCT03778229) [54,55]. In NSCLC, the H1094Y variant has just been reported as a secondary resistance variant; however, the Chilean NSCLC patients studied here exhibited this variant before any therapy indication (Figure 3A), so it is possible that H1094Y was the initial driver variant involved in the carcinogenesis of these patients.

The other novel actionable variant, which showed the highest increase in c-Met activity during proliferation and migration, was T992I, which has been previously reported as germinal in 4.5% of colon cancers, being considered an inherited risk factor for familial colorectal cancer [56]. Other studies described T992I as a rare single-nucleotide polymorphism not relevant to oncogenesis [57]. However, this variant could be relevant in NSCLC progression, since in this study we found that this variant had the highest increase in Met activity, proliferation in non-tumor cells, and migration in tumor cells, in comparison to *METex14* and H1094Y.

The study of these variants in South American patients also demonstrates the necessity of implementing NGS for NSCLC patients in Chile as a universal health policy because, until now, the clinical guides (Health Problem AUGE N°81) from Chile have mentioned that this diagnosis and treatment are highly recommended, but their high cost and lack of available financing constitute a barrier for patients [58]. Nonetheless, in the Chilean private healthcare system, some hospitals offer sequencing analysis for the diagnosis of lung cancer.

As already-approved drugs such as savolitinib, capmatinib, and crizotinib showed a decrease in the viability of cells in culture cells, the next step would be to assess the efficacy of c-Met inhibitors in patients with these variants and without other known actionable variants, as recently tested in a patient with Met H1094Y [59]. An example of NGS diagnosis and targeted therapy indication for patients with predicted actionable variants is the DRUP (Drug Rediscovery Protocol) project, a nationwide Dutch program that treated patients with any advanced solid cancer with off-label precision drugs, considering a diagnosis of actionable and bioinformatic-predicted actionable variants as biomarkers for those patients who were excluded from targeted therapies due to the absence of known actionable variants [60]. Thus, to expand the use of already-approved targeted drugs, validating the use of c-MET inhibitors against novel actionable variants is a must.

## 4. Materials and Methods

### 4.1. South American NSCLC Cohort

The cohort was selected from the study Characterization and Validation of Molecular Diagnostic Technologies for LC Patients from Chile, Brazil, and Peru (NCT03220230). The recruitment period was between July 2015 and October 2018, encompassing 37 health centers from three countries. A complete description of the study protocol has been provided previously [37,38].

### 4.2. Sequencing and Quality Control

Two to four 5 μm Tbx FFPE sections with at least 5% tumor tissue were included. The RecoverAll extraction kit (#AM1975 Thermo Fisher Scientific, Carlsland, CA, USA) was used to isolate RNA and DNA. The Oncomine Focus Assay (OFA, Thermo Fisher Scientific, Carlsland, CA, USA) was used to prepare the libraries, and they were sequenced in the Ion Personal Genome Machine System to perform NGS. The QC metric thresholds were at least 240 median reads per amplicon, 60% aligned reads for DNA libraries, 20,000 correctly mapped reads, and three out of five expression control amplicons detected for RNA.

### 4.3. Alignment and Calling of Variants

Minimum allele frequencies of 5% (SNVs) and 7% (Indels) were determined, and the minimum coverage that admits a variant was 10× (SNVs and Indels). In addition, the minimum coverage of the variant location was 50×, with the minimum variant scores in the Phred-scaled values set at 6 for SNVs and 20 for Indels. All the remaining reference/reference sites, variants with an allelic frequency < 5%, and observed alternative alleles with <10 reads were removed from the DNA VCFs. For the RNA VCFs, only fusions with more than 20 reads were maintained. Oncomine variants were selected as those located in positions within the predefined hotspot list of Oncomine Focus DNA Hotspots v1.4.

### 4.4. VUS Driver Variant Prediction

The clinical significance of the variants was obtained through categorization using Annovar (v2019Oct24) [61], the Cancer Genome Interpreter [30], and OncoKB [40]. Then, to annotate the functional consequences of the genetic variation in VUSs, we used diverse predictor algorithms such as the Cancer Genome Interpreter (CGI) platform’s prediction algorithm to identify the driver variants that could potentially be treated with targeted therapies using BoostDM and OncodriveMut [29,30]. The Combined Annotation-Dependent Depletion (CADD) value is a pathogenicity predictor that integrates several genomic features and functional predictions [31]. The SIFT algorithm predicts whether an amino acid substitution will likely affect protein function [32,33]. Polyphen-2 is another variant effect predictor that divided the analysis into three categories: benign, possibly damaging, and probably damaging to the protein function [34]. Finally, Mutation Taster was also used [62]. These predictors are integreated into Annovar (v2019Oct24). After obtaining all the algorithm’s responses, the predicted driver variants were those confirmed as predicted drivers by all the algorithms (Table S3).

### 4.5. Culture Cells

H1993, HEK293T, and BEAS-2B were maintained in RPMI 1640 (#22400089 Gibco, Carlasland, CA, USA) supplemented with 15% fetal bovine serum (SBF, #12103C, Sigma, Saint Louis, MO, USA), DMEM high glucose (#12430054, Gibco Carlasland, CA, USA) supplemented with 10% SBF, and the BEGM bronchial epithelial cell growth medium Bullet Kit (#CC-3170, Lonza, Cambridge, MA, USA), respectively, all with 1% penicillin/streptomycin (#15140122, Thermo Fisher Scientific, Carlsland, CA, USA) in a CO_2_ incubator at 37 °C. The cells were routinely tested for mycoplasma.

### 4.6. Plasmid Expression

METex14 (pLV[Exp]-Puro-CMV (hMET[NM_001127500.3]*(delete exon 14)) (VB210712-1387jhm), T992I (pLV[Exp]-Puro-CMV (hMET[NM_000245.4]*(T992I)) (VB210720-1190man), and H1094Y (pLV[Exp]-Puro-CMV (hMET[NM_000245.4]*(H1094Y)) (VB210720-1189kqp) were designed using Vector Builder (Chicago, IL, USA), and the pLentiCMV MET GFP Puro (Addgene #37560, Watertown, MA, USA) was acquired in Addgene. Dr. Marcelo Ezquer generously donated the transfection control GFP (pSIH1-H1-copGFP) vector. Lipofectamine 3000 was used in a proportion of 1:3 (DNA/lipofectamine) with the Opti-MEM™ I Reduced Serum Medium (Gibco #31985070, Carlsland, CA, USA) as a vehicle, and after 48 h, the cells were microphotographed in a Cytation3 (Agilent, Santa Clara, CA, USA), and incubated with puromycin (1 μg/mL) for 16 h. The cells were allowed to expand for 2 to 5 days before carrying out the experiments.

### 4.7. Drugs

Crizotinib (Cayman #12087, Ann Arbor, MI, USA), capmatinib (Cayman, #INCB 28060), and savolitinib (Cayman #33332) were used in quantities of 140 nM, 130 nM, and 40 nM, respectively, for 48 h, and after a total of 72 h, the MTS assay was carried out to measure the cell viability.

### 4.8. Western Blotting

The total proteins were extracted with a lysis buffer, 4× Laemmle buffer (#1610747, BIO-RAD, Berkeley, CA, USA), including a cocktail of protease and phosphatase inhibitors and 0.5 M dithiothreitol (DTT). The proteins were resolved using SDS-PAGE, transferred to the PDVF membranes, blocked with 5% bovine serum albumin (BSA, Sigma #A9418) in 0.1% TBS-Tween20, and blotted with specific antibodies: MET total (Anti-Rabbit, #ab5662, ABCAM, Waltham, MA, USA), Anti-Metp (phosphorylated on Y1230 + Y1234 + Y1235) antibody, #ab137654), anti-AKT (#4691, Thermo Fisher Scientific, Carlsland, CA, USA) and phosphorylated anti-AKT (#9271, Thermo Fisher Scientific), and β-actin (#A3853, Merck, Darmstadt, Germany). The primary antibodies were prepared in a blocking solution, and the membranes were incubated all night at 4 °C. Then, they were washed in TBS-Tween20 and further incubated with HRP-conjugated secondary antibodies. The samples were detected with EZ-ECL and visualized in an Amersham Imager 600 (GE Healthcare, Chicago, IL, USA).

### 4.9. Survival of Proliferative Cell Assay

The colorimetric assay CellTiter 96^®^ AQueous One Solution Cell (Promega #G3582, Madison, WI, USA) was used to determine the number of viable cells in proliferation using 20 μL of tetrazolium compound for 4000–5000 cells [3-(4,5-dimethylthiazol-2-yl)-5-(3-carboxymethoxyphenyl)-2-(4-sulfophenyl)-2H-tetrazolium, inner salt; MTS in 200 μL of final solution was incubated for 2 h, protected from the dark, and the absorbance at 490 nm was recorded using the Cytation3 absorbance reader (Agilent, Santa Clara, CA, USA). Then, to graph the results, the absorbances were normalized.

### 4.10. Wound Healing Assay

Twenty-four hours after seeding the already-transfected cells (empty GFP, METex14, T992I, and H1094Y) in 12-well plates with reduced fetal bovine serum, the cells were treated with and without 50 ng of recombinant human HGF (R&D #294-HG, Minneapolis, MN, USA), and the wound was immediately made with a pipette tip of 200 µL. Using the Cytation3 microscope mode (Agilent, Santa Clara, CA, USA), ten measures were taken for each wound, and the percentage of the wound closure was calculated relative to the wound distance average, at time zero, for each experimental condition.

### 4.11. Spheroid Formation

First, the spheres were performed individually in a 96-well round plate that was previously precoated with the anti-adherent Poly(2-hydroxyethyl methacrylate) (pHEMA) stock solution at 120 mg/mL, diluted in 95% ethanol PA, and allowed to homogenize while stirring overnight at room temperature, protected from light. Subsequently, on the same day of use, a working solution was created at a concentration of 5 mg/mL to incubate the 96-well, round-bottom plates. Then, the plates were incubated for 72 h at 37 °C or until the polymer was dry and translucent. Once dry, the plates were sterilized under UV light for 15 min, sealed with Parafilm, and then kept at 4 °C upside down. Next, after stabilization at room temperature, 3500 H1993 cells were seeded, which was the optimal number of cells as it ensured that the sphere did not exceed 500 µm in diameter when using the cell homogenate technique with 100 µL of RPMI. The spheroid’s size was measured with the Gen5 v3.14 Cytation3 software, taking the mean of three different diameters per sphere. The 3 independent experiments were performed using five spheres (15 spheres in total) for each experimental condition.

### 4.12. Statistical Analysis

All the statistical analyses were performed with at least three independent experiments. Statistical differences between the two conditions were assessed with a non-parametric *t*-test (Mann–Whitney correction). Comparisons between more than two conditions were analyzed with a parametric one-way or two-way ANOVA (Tukey correction for multiple comparisons). The analyses were performed with the GraphPad Prism 6 Software (San Diego, CA, USA).

### 4.13. Molecular Docking and Simulations

Molecular docking was utilized to investigate the molecular foundation and potential biological effects of MET and savolitinib (SLB). We chose to examine the segment of MET tyrosine kinase that is involved in drug interactions, based on the documented functioning MET tyrosine kinase structures found in PDBid: 8AU3, and 8AU5 [63]. We developed five separate systems for this method: (a) WT, (b) WT:SLB, (c) T992I:SLB, (d) H1094Y:SLB, and (e) METex14:SLB.

The protein structures for systems (a)–(d) were modeled in 3D by extracting amino acid residues 963 to 1345 from the entire MET sequence using AlphaFold2 v2021 [64]. These structures closely matched the secondary structure of PDBid: 8AU3 and 8AU5 [63]. We extracted the sequence segment corresponding to the original MET tyrosine kinase (1010–1345) in the case of Metexon14 and appended the preceding 70 amino acids to create a model of a similar size. Savolitinib (SLB)’s structure was obtained from Drugbank [65] (DB12048) and used as a ligand in the docking simulation process. The grid was positioned at residue H1094 to serve as a reference point for other medicines in experimental structures, resulting in a final size of 12 × 12 × 12 Å^3^. The Glide tool v4 from the Schrodinger Schrödinger suite version 2019–2 program was utilized to conduct docking simulations in the Extra Precision (XP) mode, as described by Friesner et al. in 2006 [66]. After identifying the possible starting location of SLB, the structure was evaluated to establish 5 molecular systems. The Protein Preparation Wizard in Schrödinger Suite 2023-4 was utilized to create the first configuration of the kinase enzyme structures. This involved incorporating hydrogen atoms, determining bond ordering, constructing rotamers, and assigning protonation states. Epik was utilized to predict the ionization and tautomeric states. The systems were hydrated using the TIP3P water model and subsequently balanced with a 0.15 mol/L NaCl solution, resulting in a final size of approximately 70 × 70 × 90 Å^3^. An isothermal–isobaric ensemble was established at 300 K. Each system underwent production simulations lasting 500 nanoseconds. Molecular dynamics simulations were performed utilizing the DESMOND software v2023.4 with the OPLS 2005 force-field model.

## Data Availability

The Genie data are available through The AACR Project GENIE Consortium at cBioportal. The data supporting the findings of this study are available within the article and its Supplementary Materials.

## Supplementary Materials

The following supporting information can be downloaded at https://www.mdpi.com/article/10.3390/ijms252413715/s1.
