# Supplementary material for: MET Exon 14 Skipping and Novel Actionable Variants: Diagnostic and Therapeutic Implications in Latin American Non-Small-Cell Lung Cancer Patients
Source: Int J Mol Sci. 2024 Dec 22;25(24):13715. doi: 10.3390/ijms252413715 (PMC11677537; doi:10.3390/ijms252413715)
Supplement: Supplementary file 1 [file ijms-25-13715-s001.zip › new supp Figure captions_R1.pdf]

Supplementary figure captions.

Figure S1. Distribution of the age and variant count of the subgroup of patients with MET variants. **A)** Age distribution among the patients with tumor MET variants. **B)** Distribution of patients with one or more variants.

Figure S2. A) Pearson correlations between DNA lectures with the RNA**. B)** the Allele frequency of the DNA variants with the RNA reads of the high RNA reads group.

Figure S3. Percentage of altered genes accompanying the T992I and H1094Y in the tumor mutational profiles of NSCLC patients from South America

Figure S4. Controls of transfection with GFP empty and MET wt. The H1993, BEAS2B, and the HEK293T were transfected with lipofectamine 3000, and 48 and 24h later, the transfected cells were selected with puromycin (1ug/ml) for 16h. After 72 and 48 h of growth, the tumor and non-tumor cells, respectively, were photographed in citation v3 to estimate the percentage of transfection using the GFP fluorescence of GFP alone and METwt as an approximation to standardize the transfection of a 12,990 bp plasmid.

Figure S5. Protein expression in BEAS-2B cells. **A)** Representative images of the western blot of Met total, Akt total, β-actin, Met p(Y1230-1234-1235), and Akt p(S473). **B)** The total Met expression was quantified by scanning densitometry analysis and normalized relative to β-actin expression. Graphs represent the normalized average from 3 independent experiments ±SEM. **C-D)** densitometry levels of Met phosphorylation and Akt phosphorylation normalized relative to β-actin expression. The results were normalized relative to the total Met and Akt previously normalized to actin levels (Metp/β-actin)/(Met total/ β-actin) and (Aktp/β-actin)/(Akt total/ β-actin) ±SEM. Graphs represent the normalized average from 3 independent experiments ±SEM. *p<0.05; **p<0.01; ***p<0.001.

Figure S6. Dose-response curve. Estimation of IC-50 for Crizotinib (A), Capmatinib (B), and Savolitinib (C) after 48 and 72h of treatment in 2D-HEK293T^{METex14} cells. To quantify the absorbance, the CellTiter 96® AQueous One Solution Cell was used as the datasheet indication for 5.000 cells. The cells 0 nM were incubated with the vehicle (0.01% DMSO diluted in DMEM) of the drugs.

Figure S7. Met interactions with savolitinib studied using molecular dynamic simulations. The specific contact time is described for A) MetWT:savolitinib, B) MetT992I:savolitinib and C) MetH1094Y:savolitinib systems. Protein-ligand interactions are divided into four types: Hydrogen Bonds (green), Hydrophobic (purple), Ionic (magenta) and Water Bridges (blue). The stacked bar charts are normalized over the course of the trajectory: for example, a value of 0.7 suggests that 70% of the simulation time the specific interaction is maintained.
