# Supplementary figures and images for: MET Exon 14 Skipping and Novel Actionable Variants: Diagnostic and Therapeutic Implications in Latin American Non-Small-Cell Lung Cancer Patients
Source: Int J Mol Sci. 2024 Dec 22;25(24):13715. doi: 10.3390/ijms252413715 (PMC11677537; doi:10.3390/ijms252413715)

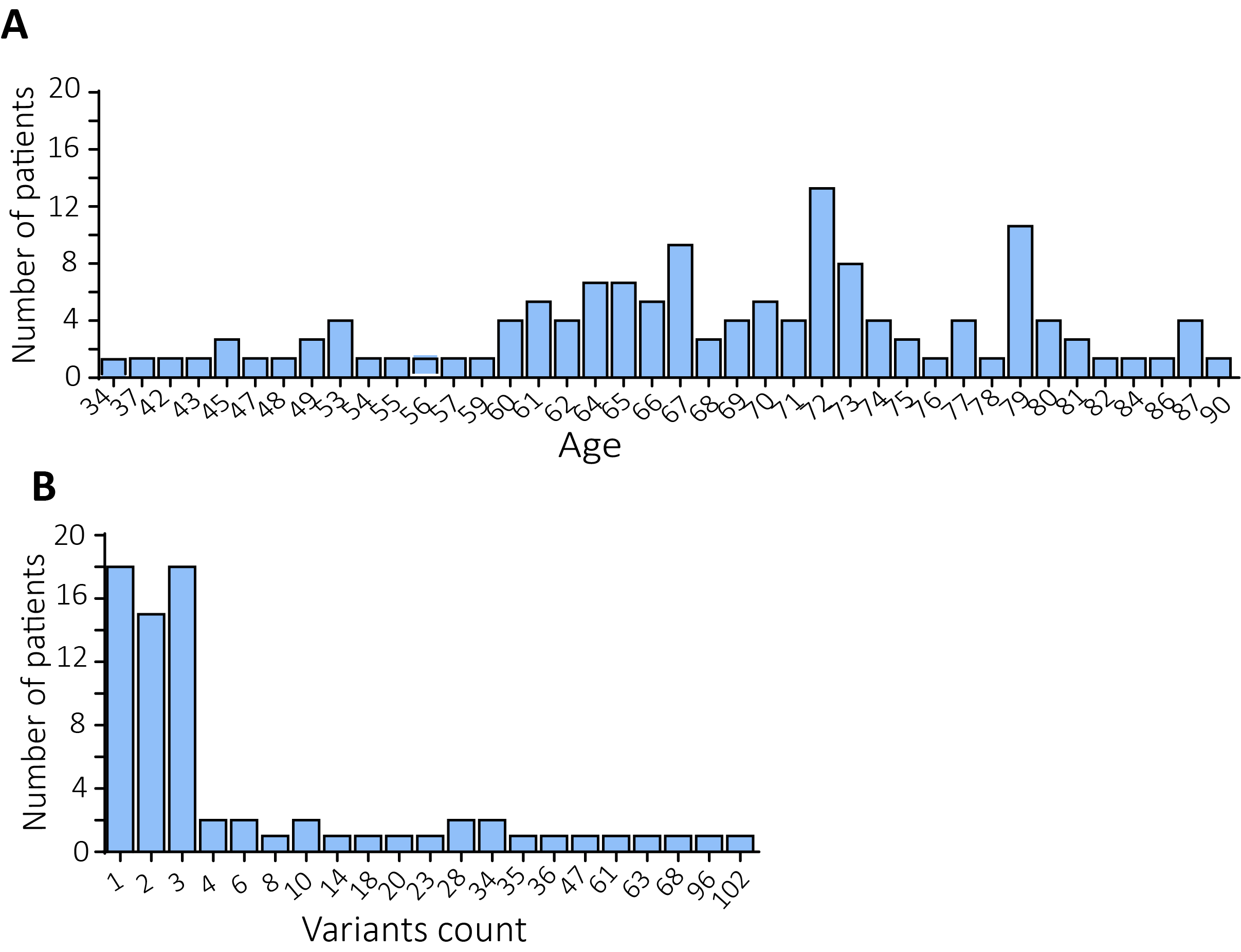

Supplement: Supplementary file 1 [file ijms-25-13715-s001.zip › Supplementary Figure_S.1.png]

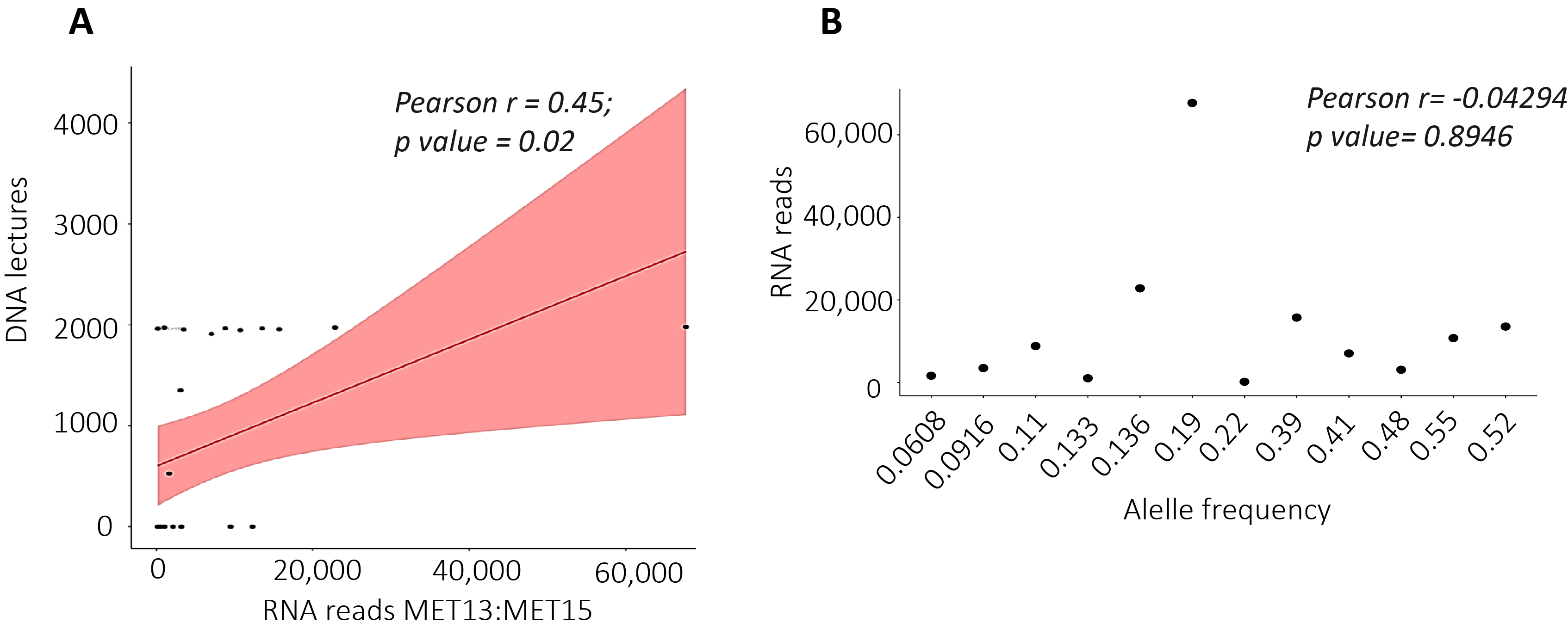

Supplement: Supplementary file 1 [file ijms-25-13715-s001.zip › Supplementary Figure_S2.png]

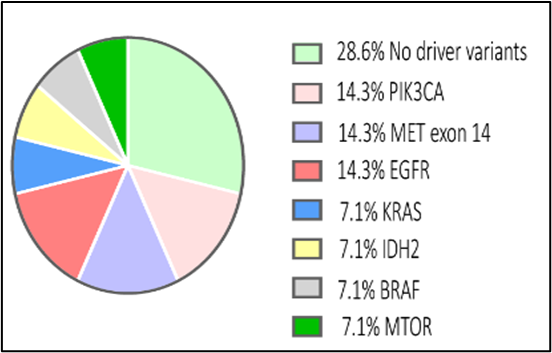

Supplement: Supplementary file 1 [file ijms-25-13715-s001.zip › supplementary Figure_S3.png]

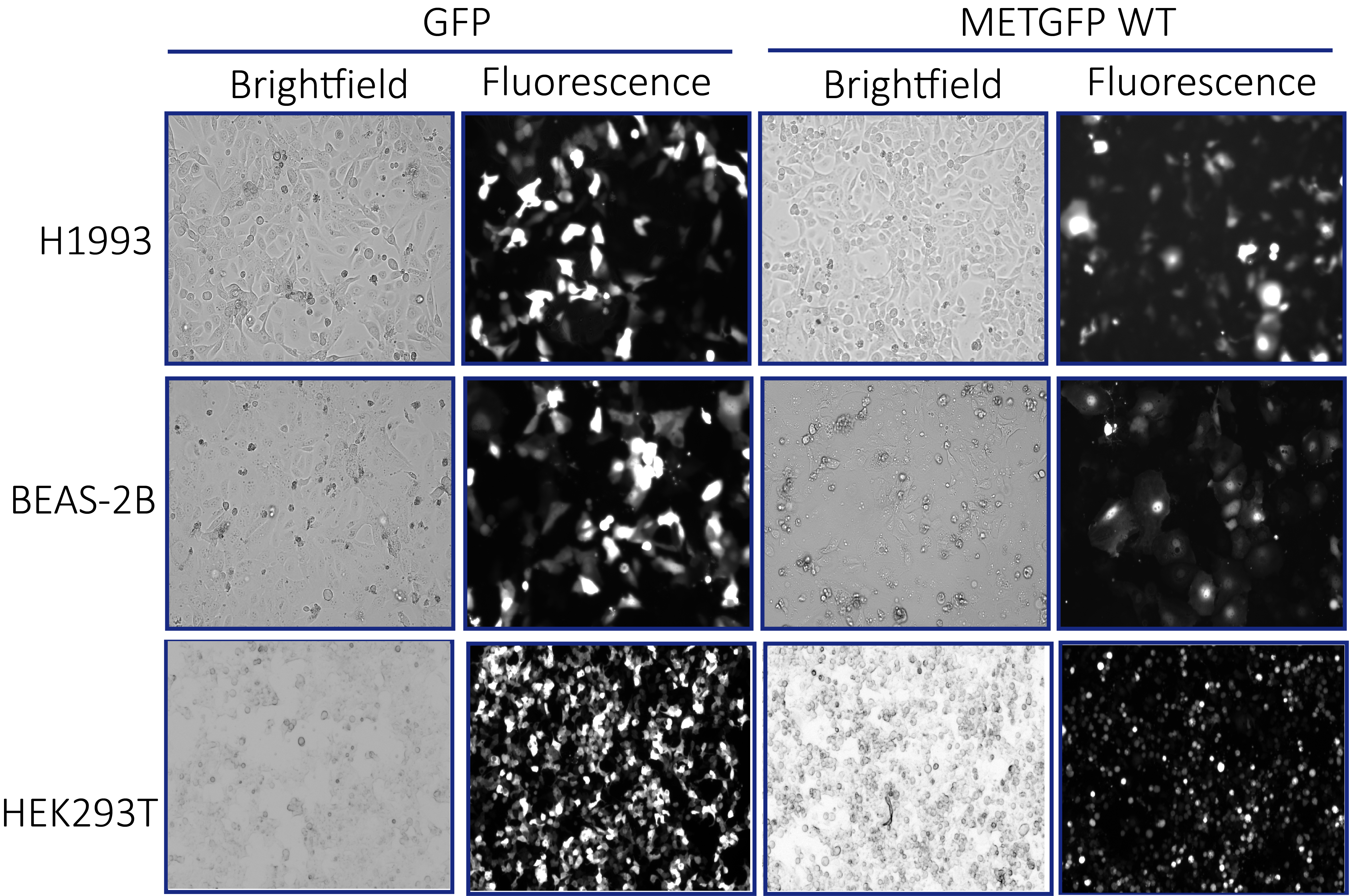

Supplement: Supplementary file 1 [file ijms-25-13715-s001.zip › Supplementary Figure_S4.png]

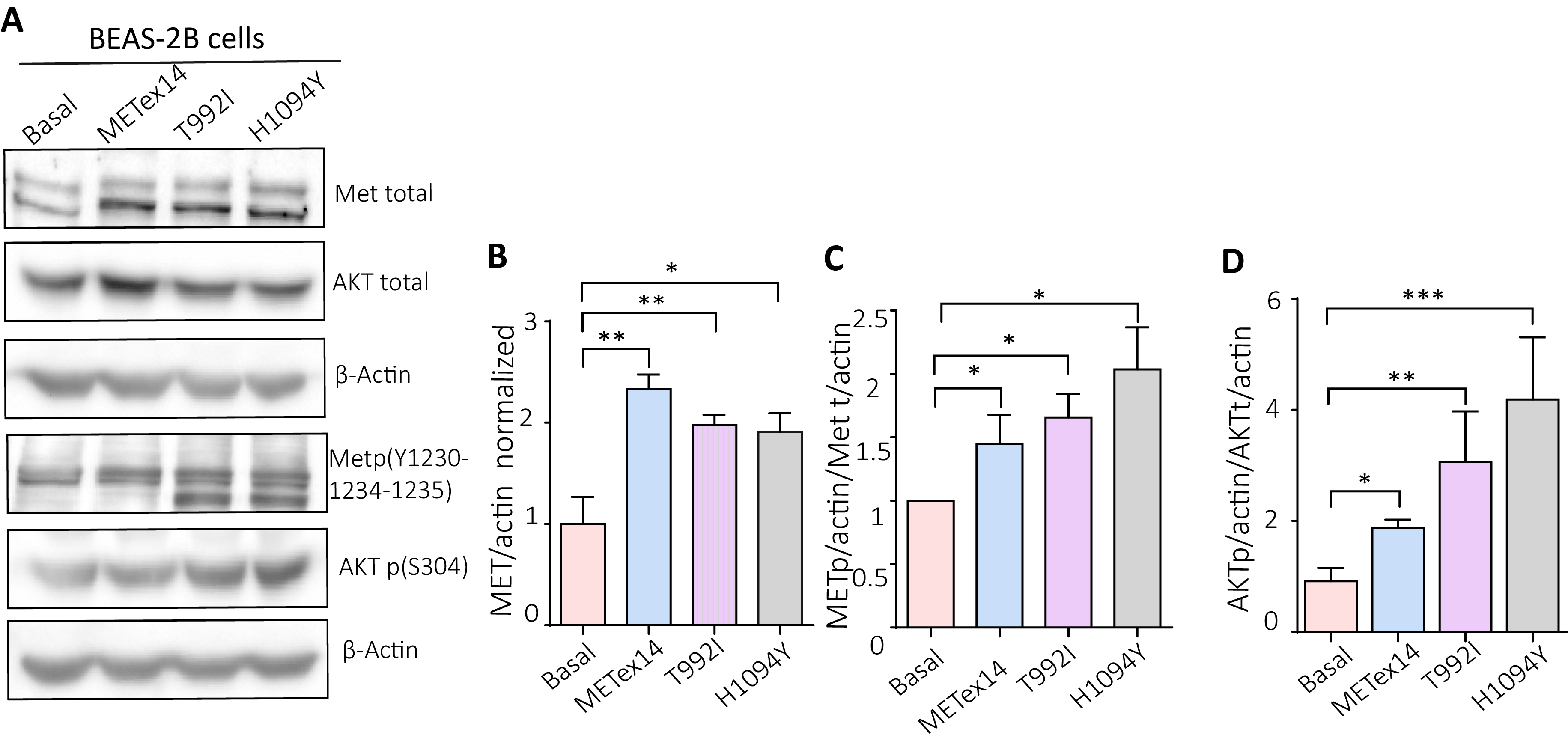

Supplement: Supplementary file 1 [file ijms-25-13715-s001.zip › Supplementary figure_S5.png]

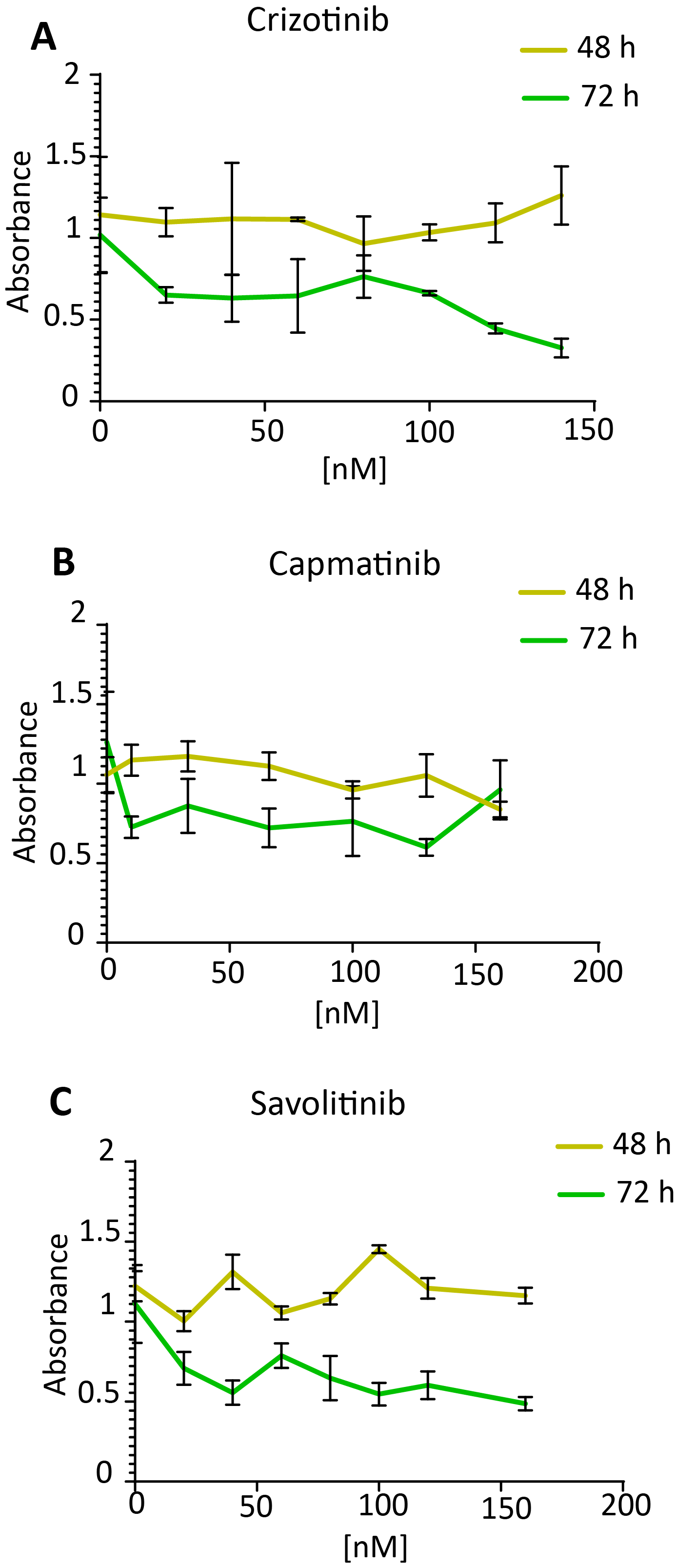

Supplement: Supplementary file 1 [file ijms-25-13715-s001.zip › supplementary figure_S6.png]

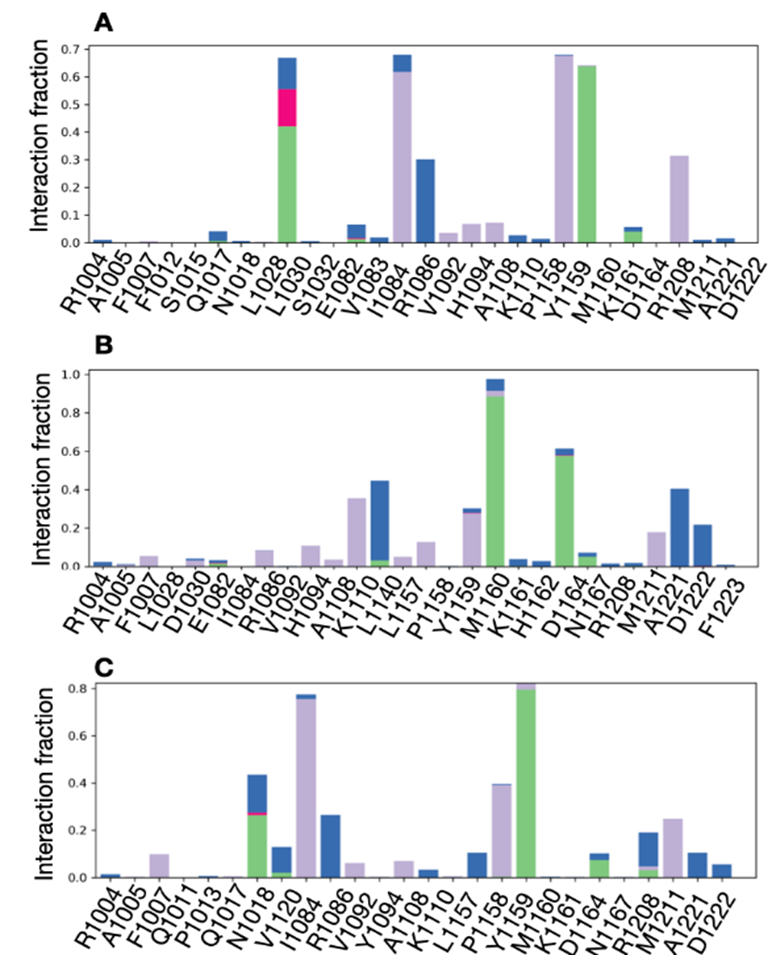

Supplement: Supplementary file 1 [file ijms-25-13715-s001.zip › Supplementary figure_S7.png]
